# Unmasking Malaria: Microscopy and nPCR Reveal the Hidden Misdiagnosis of *Plasmodium* Infections Among Febrile Pediatric in Northwest Ethiopia

**DOI:** 10.1155/ipid/9983415

**Published:** 2026-02-25

**Authors:** Sisay Getie, Gebeyaw Getnet Mekonnen, Ligabaw Worku, Meseret Birhanie, Aline Lamien Meda, Aberham Abere, Harald Noedl

**Affiliations:** ^1^ Department of Medical Parasitology, School of Biomedical and Laboratory Science, College of Medicine and Health Science, University of Gondar, P.O. Box 196, Gondar, Ethiopia, uog.edu.et; ^2^ Institute for Specific Prophylaxis and Tropical Medicine, Medical University of Vienna, Vienna, Austria, meduniwien.ac.at

**Keywords:** microscopy, misdiagnosis, Northwest Ethiopia, nPCR, pediatric, *Plasmodium*, prevalence, risk factors

## Abstract

**Background:**

Pediatric populations in Sub‐Saharan Africa (SSA) are particularly susceptible to malaria, contributing significantly to malaria‐related mortality. Accurate diagnosis of *Plasmodium* infections is crucial for early detection and prompt treatment. Malaria diagnosis relies on the use of microscopic and malaria rapid diagnostic test (mRDT) for the detection and identification of *Plasmodium* species. However, the performances of diagnostic methods are affected by epidemiology, study population, level of parasitemia, *Plasmodium* spp., and practical skills. This study aimed to assess the prevalence of *Plasmodium* infections. A diagnostic approach of microscopic and nested PCR reveals misdiagnosis of *Plasmodium* infections among febrile pediatric in Northwest Ethiopia.

**Methods:**

An institution‐based cross‐sectional study was conducted among 302 malaria‐suspected participants from March to October 2015 in Northwest Ethiopia. Capillary blood samples were collected from each study participant to detect *Plasmodium* infections, and a structured questionnaire was administered to gather data on associated risk factors. Data were analyzed using SPSS Version 23. Chi‐square test and binary logistic regression analyses were used to compare categorical variables and assess risk factors. A *p* value < 0.05 was considered statistically significant.

**Results:**

The prevalence of malaria was found to be 22.2% and 18.2% by microscopy and nPCR, respectively. Among those diagnosed by nPCR, 19.2% were males and 17.3% were females, with the highest prevalence (25.6%) observed in pediatric aged ≥ 5 years. Age, not using bed nets, and awareness of signs and symptoms of malaria were significantly associated with *Plasmodium* infections (*p* < 0.05).

**Conclusion:**

This study emphasizes the complex challenges of malaria infections in pediatric populations, worsened by restricted access to universal health coverage, which impedes efforts to reduce new infections. Furthermore, immediate actions should prioritize enhancing routine microscopic practical skills, regular quality control, and strict adherence to standardized operating procedures (SOPs) for slide preparation and examination.

## 1. Background

Malaria is a protozoan parasitic disease of humans caused by *Plasmodium species,* namely, *Plasmodium falciparum, Plasmodium vivax, Plasmodium ovale, Plasmodium malariae*, and *Plasmodium knowlesi* [1]. Malaria remains a major public health challenge globally, especially in low‐ and middle‐income countries [2]. According to the 2023 World Malaria Report, Africa accounts for approximately 94% of global malaria cases. Ethiopia is among the top contributors alongside Nigeria, the DRC, Uganda, and Mozambique [3]. Although the global trend of malaria cases and deaths is reducing due to the implementation of access to universal health coverage [4], pregnant women and pediatric are facing lion’s share of challenges, with the majority of them being from Sub‐Saharan Africa (SSA) [1]. Pediatric populations under the age of 14 were highly affected, with those under 5 years old being the most vulnerable [5]. In addition, a study conducted in Northwest Ethiopia indicated that school‐aged pediatric contributed the majority of the disease burden and accounted for the highest prevalence rate [6]. Malaria, if left untreated, leads to severe clinical and economic consequences [7]. However, malaria is a highly manageable and curable disease when diagnosed and treated early. Effective prevention and control among pediatric in Ethiopia can be achieved by addressing factors such as irrigation practices, shared housing for cattle and humans [8], insufficient household coverage of insecticide‐treated bed nets (ITNs), residency [8], and barriers that delay seeking timely treatment [9]. The common diagnostic methods of malaria in Ethiopia, such as rapid diagnostic tests (RDTs), are deployed close to the outskirts of every community at the health postlevel, whereas the microscopy method is found at the health center and hospital level [10]. In endemic countries, microscopic diagnosis continues to be considered the “gold standard” for malaria diagnosis. This method has a sensitivity of 50–500 parasites/μL, is inexpensive, and allows the identification of species and parasites [11]. In high transmission areas, microscopy is reported to detect about 75% of malaria infections, whereas in low transmission areas, however, similar approach has been shown to miss up to 88% of infections [12]. Furthermore, the level of expertise of technicians, quality of the equipment, and workload may lead to inaccurate estimates of parasite density and species differentiation. Apart from morbidity and mortality, it halted long‐lasting socioeconomic impacts primarily on more than 80% of the country’s rural community [13]. Alternatively, PCR is a highly sensitive technique, detecting low parasitemia cases missed by other techniques and easily reproducible, although it is highly expensive and time‐ and labor‐consuming [14]. This study aims to assess the prevalence of *Plasmodium* infections through a diagnostic approach of microscopic and nested PCR (nPCR), highlighting the importance of accurate malaria detection for early treatment.

## 2. Methods

### 2.1. Study Design, Period, and Setting

**Figure 1:**
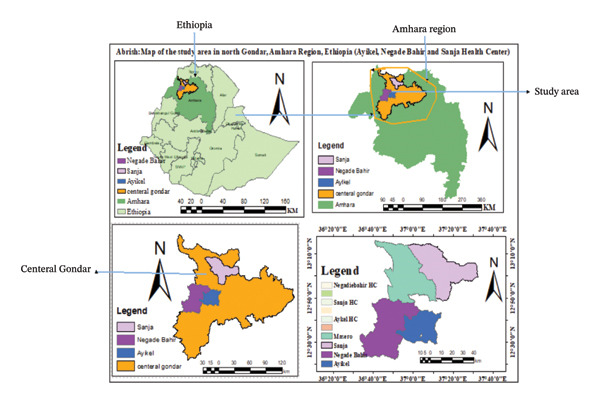
Map of the study area in North Gondar, Amhara Region, Ethiopia. Source: Maps created using shape files from GADM. Licensed under https://gadm.org/license.html.

An institution‐based cross‐sectional study was conducted from March to October 2015 at Chilga (Ayikel and Negade Bahir) and Sanja Health Center, Northwest Ethiopia, to assess the prevalence of malaria and compare the performance of microscopy and nPCR to detect *Plasmodium* infections among malaria‐suspected pediatric. These facilities are found in Northwest Ethiopia within a radius of 100 km from Central Gondar, Amhara, Ethiopia. Nearly 100 thousand people were expected to get health services at these health facilities. According to the district, the areas have an average altitude of 1500–1800 m above sea level, with annual rainfall ranging from 800 to 1800 mm and an annual temperature ranging from 25^∘^C to 42^∘^C (Figure 1) [15].

### 2.2. Study Population and Subjects

All malaria‐suspected pediatric patients attending the pediatric outpatient department (OPD) at the study site who were available during study period.

### 2.3. Inclusion and Exclusion Criteria

#### 2.3.1. Inclusion Criteria

Febrile pediatric patients aged ≤ 15 years old who had clinical signs and symptoms of malaria infection were included, and those whose parents gave written informed consent to participate in the study were included. Individuals who are permanent residents of the area and lived at least 6 months or more were included.

#### 2.3.2. Exclusion Criteria

Pediatric patients taking antimalarial treatment 2 weeks before the data collection period, severely ill, and those who had any chronic illness were excluded from the study. In addition, those who were unable to respond were excluded from the study.

### 2.4. Sample Size and Sampling Technique

#### 2.4.1. Sample Size Determination

The total sample size was determined by the single population proportion formula using a prevalence of 73.1% [16], 95% confidence level, and 5% margin of error. Therefore, the final sample size was 302.

(1) n=Zα22∗p1−pd2,

where *n* = the initial sample size, *Z α*/2 = standardized normal distribution value for the 95% CI = 1.96, *P* = proportion of *Plasmodium* infections in febrile pediatric (73.1%), and *d* = margin of error 5%.

(2) n=1.962∗0.73110.731−0.052=302.

#### 2.4.2. Sampling Technique

The total sample size was proportionally allocated to the three purposively selected health centers based on the number of suspected patients served at the laboratory for malaria diagnosis during the 10 days before the start of data collection. Therefore, 81 data from Ayikel, 119 from Negade Bahir, and 102 data from Sanja health centers were collected, respectively. Finally, proportionally allocated malaria‐suspected patients who fulfilled the inclusion criteria were conveniently recruited in the study until the allocated sample size was obtained in each of the three health centers from March to October 2015.

### 2.5. Operational Definition

 Uncomplicated *Plasmodium* Infection: According to the WHO 2015 malaria treatment guideline, uncomplicated malaria is defined as symptomatic malaria parasitemia with no signs of severity and evidence of vital organ dysfunction [17].
 Severe malaria: Acute malaria caused by *P. falciparum*, *P. vivax*, or by both species with one or more signs of organ dysfunction and high level of parasitemia (≥ 100,000 parasites/μL of blood) [18].
 Level of parasitemia: The number of asexual stages of the *Plasmodium* parasites per microliter of blood of the malaria‐infected individuals [17].
 Asymptomatic *Plasmodium* infection: A person with no recent history of clinical signs and symptoms of malaria infection who shows laboratory confirmation of parasitemia is defined as asymptomatic *Plasmodium* infection [19]. An infection is considered asymptomatic when the individual’s body temperature is < 37.5°C at presentation, no history of fever for the past 72 h, and is malaria positive by microscopy or PCR [20].

### 2.6. Data Management and Analysis

#### 2.6.1. Questionnaire Survey

The questionnaire, which includes sociodemographics and other associated factors for malaria infection, was developed in the English language after reviewing previous literature and translated into the local language (Amharic). The questionnaire was pretested on 5% of the study participants before the study began. Then, questionnaire data were collected by the trained data collector via a face‐to‐face interview technique, in parallel with capillary blood sample collection. The interview and clinical data were collected at the pediatrics OPD during a physical examination by the staff who performed the examination. Before collecting data, the data collectors identified patients who met the inclusion criteria and obtained their consent or assent. This approach aimed to minimize the nonresponse rate among study participants.

### 2.7. Blood Sample Collection and Microscopic Parasite Detection

A trained laboratory technologist collected 200 μL of capillary blood in heparinized hematocrit tubes using standard sample collection protocols from each patient for molecular analysis from each study participant to prepare both thick and thin blood films using the prelabeled microscopic slide for the detection and quantification of the *Plasmodium* parasite. Then, the thin film was fixed with methanol, and both thick and thin blood films were stained with 10% fresh Giemsa stain solution for 10 min. Then, the smears were stained with fresh Giemsa stain, and Giemsa‐stained thick and thin blood films were examined after the stained slides were air‐dried at a magnification of 100× to identify the parasite species (both asexual and gametocyte stages) and to determine the parasite density [21]. Parasite densities were determined against 200 leukocytes, assuming a standard mean white blood cell count of 8000 leukocytes per microliter of whole blood. To quantify malaria parasites against RBCs, we count the parasitized RBCs among 500–2000 RBCs on the thin smear and express the results as % parasitemia [22].

(3) Parasite/μL of blood=Number of parasites counted×8000 patents actual WBCs count/μLNumber of WBCs counted 200,Parasites/μL of blood=Number of parasitized RBCs counted × 5000000,, patents actual RBCs count/μLTotal number of WBCs counted 100.

### 2.8. Molecular Identification of *Plasmodium* species

For molecular analysis, two blood spots were collected from each participant and transferred onto filter paper (Whatman #903, GE Healthcare) labeled with the participant’s study code and date. The filter papers were allowed to air dry to avoid any chance of contamination and were put separately in clean zip‐lock plastic bags with silica desiccant. Then, filter papers in zip‐lock plastic bags were packed with large plastic bags and transported to the University of Gondar Medical Parasitology Laboratory for storage at room temperature until further analysis.

Blood samples collected on Whatman 903 filter paper were air‐dried at room temperature and placed in individual plastic bags containing silica gel desiccants and were shipped to the Institute of Specific Prophylaxis and Tropical Medicine, Medical University of Vienna, Vienna, Austria, for molecular analysis. A modified Chelex‐based DNA extraction method using the InstaGene Whole Blood Kit was used for the extraction and purification of *Plasmodium* DNA from the blood spots on filter paper. Parasite detection and species classification were conducted for all samples using a nPCR assay, following previously established guidelines. This method targeted the 18S rRNA gene and involved two amplification rounds (N1 and N2) utilizing specific primers, Taq Polymerase, and genomic DNA [23]. The resulting products were analyzed through agarose gel electrophoresis, where expected sizes were used to confirm species identity, along with controls to ensure accuracy [24].

### 2.9. Quality Control

The reliability of the study findings was assured by implementing quality control measures during the whole process of the laboratory work. Before starting the data collection, the principal investigator gave a detailed orientation about the questionnaire and recording form for data collectors. The quality of the questionnaire was checked by a pretest with 5 malaria patients at Azezo Health Center. The quality of reagents such as Giemsa stains were checked by using the known capillary blood positive and negative samples every day before starting daily work and re‐examined blindly by a senior laboratory professional from the University of Gondar Hospital Laboratory. To ensure the quality of nPCR, quality control activities were involved, including standardization of procedures. To check the performance of the test, we used known/confirmed clinical *Plasmodium* spp.’ positive and negative controls.

### 2.10. Data Management and Analysis

The data were entered into Excel using a double‐entry system and transferred to SPSS Version 23 software for analysis. Any incomplete data on the electronic database was double‐checked with the original hard copy. Frequencies and summary statistics, such as mean, standard deviation, and percentages, were generated to describe the study variables. Binary logistic regression was used to assess the associations between dependent and independent variables. Furthermore, Pearson’s chi‐square was used to measure the statistical differences between variables. In bivariate logistic regression analysis, variables with a *p* value < 0.25 were considered as potential candidates in the multivariable logistic regression analysis. The variables were considered statistically significant in the multivariable logistic regression with a *p* value < 0.05. Finally, the study findings are presented through descriptive text and tabular summaries.

## 3. Results

### 3.1. Sociodemographic and Clinical Characteristics

A total of 302 study participants were included in this study. The majority of study participants were female, 51.7% (*n* = 156). The mean age of the participants was 5.37 (±5.07 SD) years, ranging from 0.1 to 15 years, with a majority of them falling in the age group between 1 and 5 years, 59.2% (*n* = 180). About 90% (*n* = 272) of the participants reported a history of fever within the last 24 h before they were brought to the health centers, though only 19% (57/302) of them had an axillary temperature ≥ 37.5°C during physical examination. Furthermore, 29% (*n* = 88) of them had a history of vomiting before they attended the health facilities at the study sites.

### 3.2. Prevalence of Malaria Detected by Microscopy and nPCR

The prevalence of malaria was 22.2% (95% CI: 17.9–27.2; *n* = 67) and 18.2% (95% CI = 13.9–22.8; *n* = 55) by microscopy and nPCR, respectively. The prevalence of malaria using nPCR was significantly lower compared to microscopy (*p* < 0.001, McNemar’s test). From microscopic detection, 12.6% (*N* = 38), 8.9% (*n* = 27), and 0.7% (*n* = 2) of the samples were found to be positive for *P. falciparum*, *P. vivax*, and mixed infections of *P. vivax* and *P. falciparum,* respectively. nPCR detected malaria infection in 55 samples, with 10.9% (*n* = 33), 6.0% (*n* = 18), 1.0% (*n* = 3), and 0.3% (*n* = 1) of them being *P. falciparum*, *P. vivax*, *P. ovale*, and a mixed infection of *P. falciparum* and *P. vivax*, respectively. Of the total microscopically positive samples, only 35 of them were confirmed as positive by nPCR, whereas 20 samples that were negative by microscopy were positive by nPCR. Given nPCR is more sensitive than microscopy, which has its intrinsic limitations, this study revealed that 17.2% (*n* = 52) of malaria cases were diagnosed falsely by microscopy, cumulatively as positive or negative (Table 1).

**Table 1:** Discrepancy of *Plasmodium* speciation between microscopy and nPCR among febrile pediatric in Northwest Ethiopia, 2015.

nPCR results	Microscopy results					
	*P. falciparum*	*P. vivax*	*P. ovale*	*P. falciparum* and *P. vivax*	Negative	Total
*P. falciparum*	15	3	0	2	13	33
*P. vivax*	7	6	0	0	5	18
*P. ovale*	1	0	0	0	2	3
*P. falciparum and P. vivax*	1	0	0	0	0	1
Negative	14	18	0	0	214	247
Total	38	27	0	2	235	302

The prevalence of malaria among pediatric patients who had felt fever in the last 24 h before they were brought to the health facilities was 23.2% (*n* = 63) and 17.3% (*n* = 47) by microscopy and nPCR, respectively. Whereas among subjects who were febrile during physical examination, 26.3% (*n* = 15) and 29.8% (*n* = 17) were malaria positive by microscopy and nPCR, respectively. On the other hand, among participants who had a history of vomiting, 23.9% (*n* = 21) and 21.6% (*n* = 19) of them were malaria positive by microscopy and nPCR, respectively.

In this study, microscopically detected malaria was found in 21.9% (32/146) of the male and 23.7% (37/156) of female participants, and a slightly higher prevalence (23.3%) among the age group between 1 and 5 years old. Whereas nPCR‐detected malaria was found in 19.2% (28/146) of male and 17.3% (27/156) of female study subjects, while the highest prevalence (25.6%) was observed among the age group ≥ 5 years old (*χ* ^2^ = 8.409, *p* = 0.015). On the other hand, in comparison of parasitemia among microscopically detected malaria, the higher the age beyond 5 years old tends to have low parasitemia, while the age group between 1 and 5 years old had higher parasitemia levels (*χ* ^2^ = 30.366, *p* ≤ 0.001) (Table 2).

**Table 2:** Level of parasitemia to gender, age, and *Plasmodium* species among febrile pediatric patients in Northwest Ethiopia, 2015.

Parameter		Parasite density distribution per microliter of blood					
		< 5000	5000–10,000	≥ 10,000	Total	*p* value	*χ* ^2^
Sex	Male	9	10	12	31	0.555	1.177
	Female	15	10	11	36		

Age group (years)	< 1 yr	0	7	3	10	0.001	30.366
	1–4.99 yrs	11	12	19	42		
	≥ 5 yrs	13	1	1	15		

*Plasmodium* species	*P. falciparum*	9	10	19	38	0.009	13.643
	*P. vivax*	14	10	3	27		
	Mixed	1	0	1	2		

Axillary body temperature	Febrile (≥ 37.5°C)	6	3	7	16	0.490	1.428
	Not febrile (< 37.5)	18	17	16	51		

### 3.3. Risk Factors Associated With Malaria Among Pediatric Febrile Patients

In this study, the multivariate analysis revealed that the risk of contracting malaria among pediatric is associated with certain age groups, bed net usage, lack of health education, and lack of awareness of parents on the signs and symptoms of malaria. Accordingly, pediatric patients in the age group between 1 and 5 years and greater than five years old were 5.6 times (AOR = 5.6, 95% CI: 1.01–31.1, *p* = 0.049) and 13 times (AOR = 13, 95% CI: 2.05–92.3, *p* = 0.007) more likely to develop malaria, respectively. Moreover, pediatric patients who did not use bed nets at all times were 12.35 times (AOR = 12.35, 95% CI: 4.75–32.1, *p* ≤ 0.001) more likely to be infected by malaria than those who used it daily (Table 3).

**Table 3:** Multivariate analysis of factors associated with malaria infection among febrile pediatric patients in Northwest Ethiopia, 2015.

Associated risk factors		Malaria by nested PCR		OR (95% CI)		*p* value
		Positive (%)	Negative (%)	COR	AOR	
Sex	Male	28 (19.2)	118 (81.8)	1.13 (0.63–2.03)	1.2 (0.56–2.60)	0. 62
	Female	27 (17.3)	129 (82.7)	1	1	

Age (years)	< 1	2 (4.5)	42 (95.5)	1	1	
	1–5	33 (18.3)	147 (81.7)	4.7 (1.09–20.5)	5.6 (1.01–31.1)	0.049
	≥ 5	20 (25.6)	58 (74.4)	7.2 (1.61–32.68)	13 (2.05–92.3)	0.007

Family size	≤ 3	8 (12.5)	56 (87.5)	1	1	
	4–6	32 (17.6)	150 (82.4)	1.5 (0.65–3.44)	2.1 (0.58–7.5)	0.26
	≥ 7	15 (26.8)	41 (73.2)	2.56 (1.0–6.61)	225 (0.45–10.8)	0.33

Residence	Urban	25 (18)	114 (82)	1	1	
	Rural	30 (18.4)	133 (81.6)	1.03 (0.57–1.85)	0.91 (0.38–2.17)	0.837

Parents educational status	Illiterate	38 (22)	135 (78)	1.6 (0.44–5.73)	1.55 (0.12–19.5)	0.735
	Can read and write	13 (14)	80 (86)	0.92 (0.24–3.59)	1.006 (0.10–9.9)	0.996
	Completed high school	1 (6.3)	15 (93.7)	0.38 (0.04–4.03)	0.4 (0.02–9.66)	0.573
	Completed college and above	3 (15)	17 (85)	1	1	

Parents’ occupation	Farming	42 (18.4)	186 (81.6)	1	1	
	Business	2 (9.5)	19 (90.5)	0.47 (0.12–2.08)	0.35 (0.04–3.39)	0.363
	Employee	5 (16.7)	25 (83.3)	0.89 (0.32–2.45)	1.96 (0.26–14.5)	0.512
	Others	6 (26.1)	17 (73.9)	1.56 (0.58–4.2)	2.68 (0.64–11.2)	0.177

Parents’ monthly income (approximate Ethiopian birr)	< 1000	25 (18.9)	107 (81.1)	0.935 (0.40–2.19)	1.3 (0.31–5.31)	0.725
	1000–3000	21 (16.4)	104 (83.6)	0.81 (0.34–1.92)	1.34 (0.40–4.48)	0.633
	> 3000	9 (20)	36 (80)	1	1	

House distance from water body	< 3 km	23 (23.2)	76 (76.8)	1.4 (0.78–2.78)	1.65 (0.49–5.61)	0.420
	3–5 km	12 (13.5)	77 (86.5)	0.73 (0.34–1.59)	1.5 (0.44–5.13)	0.518
	> 5 km	20 (17.5)	94 (82.5)	1	1	

Frequency of bed net usage	Daily	7 (4)	168 (96)	1	1	
	Sometimes	20 (33.9)	39 (66.1)	7.8 (3.3–18.43)	8.84 (3.35–23.35)	0.001
	Not at all	28 (41.2)	40 (58.8)	11.9 (5.35–26.48)	12.35 (4.75–32.1)	0.001

House sprayed with insecticide	Yes	6 (17.1)	29 (82.9)	1	1	
	No	49 (18.4)	218 (81.6)	1.09 (0.43–2.76)	1.15 (0.36–3.69)	0.818

Health education by HEWs	Yes	22 (14)	135 (86)	1	1	
	No	33 (22.8)	112 (77.2)	1.81 (1.0–3.28)	1.79 (1.03–4.08)	0.043

Family awareness on signs and symptoms of malaria	Yes	52 (19.3)	217 (80.7)	1	1	
	No	3 (9.1)	30 (90.9)	2.4 (0.70–8.16)	4.79 (1.06–21.66)	0.042

## 4. Discussion

Malaria is a huge public health problem in terms of morbidity and burden on healthcare facilities [25, 26]. It accounts for the increasing percentage of outpatient consultations in most health facilities in different regions in Ethiopia [27]. In this study, the prevalence of malaria among malaria‐suspected pediatric patients was 22.2% (67/302) (95% CI: 17.9–26.8) and 18.2% (55/302) (95% CI: 13.9–22.5) by microscopy and nPCR, respectively. This finding was comparable with studies conducted in Ethiopia [11, 28]. However, this result was lower than a similar study performed in Cameroon, 36.6% [29], and Kenya, 43.7% [30]. On the other hand, this finding was higher than a study performed in Metema, Northwest Ethiopia [6]. The possible reason for the difference in prevalence may be due to the variation in the type of study design used, climatological differences, altitude variation, malaria diagnosis technique variation, the skill of the laboratory personnel to detect and identify malaria parasites, and other factors that affect malaria case occurrences in different study areas [31].

The predominant *Plasmodium* species detected was *P. falciparum* (60%), followed by *P. vivax* (32.3%) and *P. ovale* at the study sites*.* The majority (98.2%) of infections were monoinfections*.* This was in agreement with previous studies conducted elsewhere in Ethiopia [32–34]. In contrast, another study reported that *P. vivax* was the most prevalent species, followed by *P. falciparum,* which indicated the shifting of *P. vivax* in the study area and different parts of Ethiopia, and no report of *P. ovale* cases recently [27, 34]. Even though the epidemiology of *P. ovale* remains poorly understood, there were similar recent data on the distribution of this pathogen in Gondar, Ethiopia [35]. However, almost all previous studies conducted in Ethiopia are based on microscopic data only, whereas this study used a highly sensitive nPCR assay with a documented limit of detection of only six parasites/μL(24). In addition, this difference might be due to altitude variation and climatological differences that may contribute to a great role in the breeding of the *Anopheles* vector and survival of the parasite in the vector [36].

The 5.5% positive rate for *P. ovale* infections among malaria cases is the 2^nd^ report of *P. ovale* from this part of the country. Until recent times, the distribution pattern of *P. ovale* was considered to be limited to tropical regions outside Ethiopia [37]. However, still, it is not from health facilities treating malaria in Ethiopia, perhaps due to its tertian periodicity, typically low parasitemia, and morphological resemblance with *P. vivax* [38]. Moreover, it might be due to difficulty in the diagnosis of *P. ovale* by microscopic examination in the study area, where this pathogen was previously not known to be present, and most microscopists are neither trained to detect nor aware of the presence of other species [39–41].

In this study finding, we also observed a statistically significant negative relationship between age and parasite density. There was a decline in *Plasmodium* parasitemia as age increased. A similar finding was reported in Papua New Guinea [42]. This may reflect the higher endemicity of the current study site, where acquired immunity against the parasite due to reinfection reduces parasite density in older age groups [43]. Fever has been the major complaint among pediatric patients presenting at outpatient clinics, with malaria being the possible etiology for such febrile illnesses [44]. In our study, a proportion of pediatric under the age of 15 with *Plasmodium* infections had only 24.7% (22/89) fever on physical examination. Similar findings have been reported from malaria‐endemic areas [45, 46]. The presence of malaria parasites without febrile illness could be explained in part by the acquired protective immunity. In endemic areas, protective immunity against malaria is acquired with an increase in exposure to malaria parasites, which increases with age [47].

In addition, other factors have been described to predict malaria‐like fever, duration of fever, intermittent fever, and malaria parasitemia, and signs and symptoms such as headache, anemia, and vomiting were also predicted [48]. However, in our study, the presence of fever or a history of fever and anemia predicts malaria. Similar findings have been reported from elsewhere [47, 49, 50]. In contrast, findings have been reported in Gambia, where the use of fever or history of fever resulted in the overdiagnosis of malaria [51]. Regarding the level of parasitemia, in this study, the level of parasitemia has a significant association with the age group, which showed that the majority of the heaviest parasitemic study participants fall in the 1–5‐year‐old age group. A similar finding was reported from elsewhere [52]. Other pediatrics who are less than 1 year and greater than 5 years old are more likely to acquire protective immunity from their mother and after repeated exposure to malaria infection, respectively [53].

In this study’s findings, we had also evaluated the risk factors associated with malaria. An association was observed between malaria and the monthly income of the family, where significantly higher, 32.4% (57/119), prevalence of malaria was found in monthly income less than 1000 Ethiopian birr than those who had a monthly income of more than 3000 Ethiopian birr, 9.1% (5/57) (*p* < 0.05). A similar result was also reported from studies conducted in Kenya [54], in four SSA countries [55], and in Africa [56]. This may be due to low socioeconomic people being more vulnerable because they are more likely than others to be homeless and sleep outside and less likely to seek preventive measures and health services. However, this study suggests that the rich are more likely to use these preventative measures to effectively counteract the spread of malaria.

In the present study, the status of malaria and bed net utilization was strongly associated. The odds of being infected with malaria were 12.35 times higher in those individuals who were not using a bed net than in those who used it daily (*p* < 0.05). This finding is supported by studies conducted in different parts of Ethiopia [57–59]. This is due to the fact that using a bed net daily reduces the risk of malaria infection by preventing human–mosquito contact. ITNs provide protection both to the individuals sleeping under them by deterring mosquito bites and to family members by killing mosquitoes, thereby reducing transmission of malaria parasites [60].

In addition, pediatric families who did not gain health education from a health worker are also 1.79 times more likely to be exposed to malaria infection than those who had gained health education. This finding is supported by studies in Colombia [61] and Southeast Nigeria [62]. This suggests that pediatric families who were well trained about the correct use of mosquito nets, antimalarial spraying, and other malaria preventative measures, coupled with factors such as the number of rooms in a house, are less vulnerable to malaria infection.

The nPCR analysis confirms a relatively high false‐positive rate of 10.6%, with 6.6% false‐negative cases at microscopy as well as misclassification of *Plasmodium* species (e.g., *P. ovale* as *P. vivax or P. falciparum; P. falciparum* as *P. vivax;* and *P. vivax* as *P. falciparum*). Even though microscopy remains the gold standard for malaria diagnosis in the field, the limits of detection may significantly differ between microscopists and have previously been estimated to range from 50 to 100 parasites/μL under field conditions [63]. The performance of microscopy depends on well‐maintained equipment, an uninterrupted supply of good‐quality reagents, trained staff, the type and quality of the smear, the skill of the technician, the parasite density, and the time spent on reading the smear. Maintaining a quality‐assured microscopy service is a major challenge, even for health centers and district hospitals [64]. Individuals who are falsely diagnosed with malaria are exposed to unnecessary side effects of drugs and underestimate the most probable cause of febrile illness among pediatric.

### 4.1. Limitations and Strengths of the Study

This study provides valuable insights by evaluating the advancements and challenges in detecting *Plasmodium* infections in real‐world healthcare settings, highlighting the current state of common diagnostic methods and their implications for malaria elimination efforts. However, several limitations should be considered when interpreting the findings. First, the study was unable to identify specific factors leading to misdiagnosis in routine microscopy, primarily due to the inclusion of a limited number of health centers and professionals, which was constrained by available resources. This limitation affected both the sample size and the ability to determine precise causes of diagnostic errors. Second, the absence of RDTs limited the study′s capacity to fully characterize submicroscopic infections. Despite these limitations, the study contributes valuable knowledge for improving malaria diagnosis and guiding strategies to strengthen diagnostic accuracy in pediatric populations.

## 5. Conclusions and Recommendations

This study highlights the multifaceted nature of malaria infections among pediatric populations, exacerbated by limited access to universal health coverage and a lack of community health awareness in Ethiopia. To address these challenges, the Ethiopian health system must align with its malaria elimination efforts, focusing on increasing the utilization of ITNs. Moreover, comprehensive training for healthcare professionals managing malaria is crucial, as inadequate services can harm pediatric health and result in poor clinical outcomes, hindering efforts to reduce new infections.

Immediate actions should prioritize enhancing routine microscopy through improved training, regular quality control, and strict adherence to standardized operating procedures (SOPs) for slide preparation and examination. The study also emphasizes the necessity for further research to bridge existing gaps in malaria diagnostics. Collaborative efforts among governments, laboratories, and health bureaus are crucial for establishing robust quality assurance programs and securing funding for effective diagnostic practices. Future research should focus on multicenter studies with larger sample sizes to better understand factors contributing to misdiagnosis and inform targeted interventions.

**Nomenclature**
ITNs: Insecticide‐treated bed nets
nPCR: Nested polymerase chain reaction
OPD: Outpatient’s department
RDT: Rapid diagnostic test
SSA: Sub‐Saharan Africa

## Author Contributions

Conceptualization: Sisay Getie, Gebeyaw Getnet Mekonnen, Ligabaw Worku, Meseret Birhanie, Aline Lamien Meda, Aberham Abere, and Harald Noedl.

Data management: Sisay Getie, Aberham Abere, and Harald Noedl.

Formal analysis: Sisay Getie, Gebeyaw Getnet Mekonnen, Aberham Abere, and Harald Noedl.

Investigation: Sisay Getie, Gebeyaw Getnet Mekonnen, Aberham Abere, and Harald Noedl.

Methodology: Sisay Getie, Gebeyaw Getnet Mekonnen, Aberham Abere, and Harald Noedl.

Software: Sisay Getie, Gebeyaw Getnet Mekonnen, Aberham Abere, and Harald Noedl.

Visualization: Sisay Getie, Gebeyaw Getnet Mekonnen, Ligabaw Worku, Meseret Birhanie, Aline Lamien Meda, Aberham Abere, and Harald Noedl.

Writing–original draft: Sisay Getie, Gebeyaw Getnet Mekonnen, Ligabaw Worku, Meseret Birhanie, Aline Lamien Meda, Aberham Abere, and Harald Noedl.

Writing–review and editing: Sisay Getie, Gebeyaw Getnet Mekonnen, Ligabaw Worku, Meseret Birhanie, Aline Lamien Meda, Aberham Abere, and Harald Noedl.

## Funding

No funding was obtained for this study.

## Disclosure

The paper was available as a preprint on research square at the following link: https://www.researchsquare.com/article/rs-4646994/v1.

## Ethics Statement

This study is an extension of an epidemiological study of malaria among pediatric patients suspected of malaria that has been ethically approved (Reference number: CMHS 278/04/08) by the Research and Ethics Review Committee of the University of Gondar, Gondar, Ethiopia. Permission was obtained from the Zonal Health Administrative and participating Health centers. In addition, after explaining the importance of the study, written informed consent was obtained from the study participant’s parent/guardian. Pediatric patients who were positive for malaria infection were handled by the respective health facility according to the malaria diagnosis and treatment guidelines of the country.

## Consent

Please see the Ethics Statement.

## Conflicts of Interest

The authors declare no conflicts of interest.

## Data Availability

Data are available upon request.
